# Stem Cell Antigen 1-Positive Mesenchymal Cells Are the Origin of Follicular Cells during Thyroid Regeneration

**DOI:** 10.1371/journal.pone.0080801

**Published:** 2013-11-21

**Authors:** Minoru Okamoto, Suguru Hayase, Masaaki Miyakoshi, Tsubasa Murata, Shioko Kimura

**Affiliations:** Laboratory of Metabolism, National Cancer Institute, National Institutes of Health, Bethesda, Maryland, United States of America; Ecole Normale Supérieure de Lyon, France

## Abstract

Many tissues are thought to contain adult stem/progenitor cells that are responsible for repair of the tissue where they reside upon damage and/or carcinogenesis, conditions when cellular homeostasis becomes uncontrolled. While the presence of stem/progenitor cells of the thyroid has been suggested, how these cells contribute to thyroid regeneration remains unclear. Here we show the origin of thyroid follicular cells and the process of their maturation to become follicular cells during regeneration. By using β-galactosidase (β-gal) reporter mice in conjunction with partial thyroidectomy as a model for thyroid regeneration, and bromodeoxyuridine (BrdU) long label-retaining cell analysis, we demonstrated that stem cell antigen 1 (Sca1) and BrdU-positive, but β-gal and NKX2-1 negative cells were found in the non-follicular mesenchymal area 7 days after partial thyroidectomy. They temporarily co-expressed cytokeratin 14, and were observed in part of follicles by day 35 post-partial thyroidectomy. Sca1, BrdU, β-gal, and NKX2-1-positive cells were found 120 days post-partial thyroidectomy. These results suggested that Sca1 and BrdU positive cells may participate in the formation of new thyroid follicles after partial thyroidectomy. The process of thyroid follicular cell regeneration was recapitulated in *ex vivo* thyroid slice collagen gel culture studies. These studies will facilitate research on thyroid stem/progenitor cells and their roles in thyroid diseases, particularly thyroid carcinomas.

## Introduction

Currently very little is known about thyroid stem/progenitor cells and their specific markers. In recent years, the presence of stem/progenitor cells in the thyroid has been suggested in mouse [1] and human [2], [3] using Hoechst dye-resistant side population (SP) cells, or with direct use of human thyroid tissues derived from patients with Goiters, and/or thyroid cell lines, as determined by qRT-PCR and/or immunohistochemistry. Further, spheroids having self-replicative potential were obtained using surgical thyroid specimens from patients with thyroid adenoma and Grave's disease [4]. These spheroids generated follicles with thyroid hormone production, while they produced progeny expressing the neuronal marker β-tubulin when co-cultured with a neuroblastoma cell line, or underwent adipogenic differentiation when cultured in adipogenic medium. None of these studies, however, identified a specific marker(s) for thyroid stem/progenitor cells.

Several models have been used to study stem/progenitor cells without the knowledge and use of a specific stem/progenitor marker(s). Among them is to treat cells with the dye Hoechst 33342, followed by dual-wavelength fluorescence–activated cell sorting (FACS). This results in a small but distinct subset of cells called side population (SP) [5]. SP cells are present in a wide variety of mammalian tissues including hematopoietic and non-hematopoietic tissues [6]–[13], and are considered to contain multipotent stem cells [9], [12], [13]. Using Hoechst dye-resistant SP cells, we previously demonstrated that ∼0.3–1.4% of total thyroid cells represented SP cells, which exhibited stem/progenitor-like characteristics in terms of gene expression and cultured cell morphology [1]. Approximately one-half of them expressed Sca1 (stem cell antigen 1), as determined by FACS, the gene originally identified as an adult murine hematopoietic stem cell marker [14], [15]. Sca1 is now widely used as a candidate marker in search of tissue-resident and cancer stem cells of various tissues [10], [16]–[18].

Another approach to study stem/progenitor cells without specific knowledge of a marker(s) is to use long label-retaining cells analysis. In this experiment, cells are pulse-labeled by a DNA precursor such as tritiated thymidine or bromodeoxyuridine (BrdU). The following prolonged chase period results in dilution of labeled cells due to active proliferation of most cells. Long label-retaining cells are hypothesized to result from either slow-cycling or asymmetric cell division that is thought to be intrinsic to the nature of stem/progenitor cells [19]–[23]. For instance, BrdU positive cells from the forebrain ventricles of adult mice are positive for a neural stem cell marker Nestin, which clonally give rise to great numbers of Nestin(+) neural precursors [24]. Further, long label-retaining cells in bladder are small, low granularity, and positive for β4 integrin, an epidermal stem cell marker, and demonstrate superior clonogenic and proliferative ability [25]. Thus, long label-retaining cells have been proposed to represent adult tissue stem cells.

This study was undertaken to understand the nature of Sca1-expressing cells and their relation to follicular cell regeneration in mouse thyroids. R26R;TPO-Cre reporter mice that express β-galactosidase (β-gal) following thyroid follicular cell-specific expression of Cre recombinase were subjected to partial thyroidectomy (PTx) as a model for thyroid regeneration in conjunction with BrdU administration for long label-retaining cell analysis. These studies identified Sca1-expressing non-follicular mesenchymal cells as the origin of follicular cells during thyroid regeneration.

## Methods

### Ethics statement

All animal studies were performed in accordance with the Using Animals in Intramural Research Guidelines (National Institutes of Health Animal Research Advisory Committee, National Institutes of Health, Bethesda, MD) and after approval of the National Cancer Institute's Animal Care and Use Committee.

### Animals

R26R;TPO-Cre mice were produced by crossing TPO-Cre [26] and ROSA26 mice that expresses β-gal upon Cre-mediated recombination of the R26R locus [27]. Details for these mouse lines and genotyping methods were previously described [26], [27]. Partial thyroidectomy (PTx) was carried out to remove caudal one third of both thyroid lobes. For long label-retaining cell analysis, BrdU (50 µg/g body weight) was intraperitoneally injected twice a day for 6 consecutive days, starting one day after PTx. Animals were subjected to histological, immunohistochemical, and/or immunofluorescence analyses on day 7, 14, 35, 75, and 120 days post-PTx.

### Histology, immunohistochemistry, and immunofluorescence

A cervical region of a mouse containing the thyroid, larynx and trachea was dissected, fixed in 10% buffered formalin at room temperature or 37°C overnight, dehydrated, and embedded in paraffin. Serial sections of 3 or 4 µm thickness were treated with xylene and graded ethanol, and then stained with hematoxyline and eosin (H&E). For immunohistochemistry and immunofluorescence, epitope retrieval was carried out using steamer (45 min in 10 mM citrate buffer, pH 7.0), autoclave (5 min in citrate buffer, pH 6.0 or 1× TE, pH 9.0), or proteinase K digestion (50 µg/ml, 37°C, 20 min). After cooling to room temperature, endogenous peroxidase activity was quenched using 0.3% H_2_O_2_ in methanol for 30 min at room temperature for immunohistochemistry. Non-specific binding sites were blocked using 10% normal goat serum or normal donkey serum (Jackson Immuno Research Lab, West Grove, PA) in PBS before incubation with a primary antibody. The sections were incubated overnight at 4°C or for 1 hour at room temperature with the following primary antibodies; anti-BrdU (rat polyclonal, 1∶100, Serotec, Raleigh, NC), anti-Sca1 (rabbit monoclonal, 1∶600, Abcam, Cambridge, MA), anti-cytokeratin (Krt) 14 (rabbit polyclonal, 1∶2000, Covance, Emeryville, CA), anti-NKX2-1 (rabbit polyclonal, 1∶100, Santa Cruz Biotechnology, Santa Cruz, CA), anti-β-gal (chicken polyclonal, 1∶1000, Abcam), anti-CD34 (rat polyclonal, 1∶25, Abcam), anti-CD133 (rabbit polyclonal, 1∶ 50, Abcam), anti-Sox-10 (goat polyclonal, 1∶50, Santa Cruz Biotechnology), anti-Oct-3/4 (mouse monoclonal, 1∶200, Santa Cruz Biotechnology), and anti-GATA4 (rabbit polyclonal, 1∶200, Santa Cruz Biotechnology).

For immunohistochemistry, the sections were washed by PBS, followed by treating with HRP-conjugated rabbit anti-rat IgG (Abcam) with MOM kit (Vector Laboratories, Burlingame, CA) or anti-chicken IgY (Abcam), or the ABC method with a commercially available kit (Vector Laboratories) according to the manufacturer's instructions. Immunostaining was visualized with 3,3′-diaminobenzidine (DAB) as substrate (Sigma, St Louis, MO), and counterstained with hematoxylin. Positive cell numbers were determined by counting whole cell numbers of a section from a thyroid lobe and were expressed as those per 1000 intrafollicular cells.

For immunofluorescence, the sections were treated with the following secondary antibodies; labeled goat anti-rabbit IgG antibodies (Alexa Fluor 555, 1∶800, Abcam, Dylight 594, 1∶1000, Thermo scientific), labeled goat anti-rat IgG antibodies (Alexa Fluor 488, 1∶200, Invitrogen, Alexa Fluor 647, 1∶1000, Abcam), labeled goat anti-chicken IgY antibodies (Dylight 650, 1∶300, Abcam), and labeled donkey anti-goat IgG (Alexa Fluor 488, 1∶200, invitrogen, Alexa Fluor 555, 1∶800, Abcam, Alexa Fluor 594, 1∶1000, Invitrogen).

For multi-stain immunofluorescence, two or three different primary antibodies or secondary antibodies were mixed, while for double labeling using two primary antibodies from the same host species, unconjugated Fab fragments were used for blocking after first secondary antibody. For instance, multi-stain (Sca1, BrdU, β-gal, NKX2-1) was performed as follows. After epitope retrieval and blocking of non-specific binding sites, sections were incubated with the first primary antibody (anti-Sca1) for 1 hour at room temperature. After washing with PBS, sections were incubated with the first secondary antibody (Alexa Fluor 555 goat anti-rabbit IgG) and washed with PBS. Sections were then incubated with normal serum (5% rabbit serum) from the same host species as the first primary antibodies for 1 hour at room temperature and washed with PBS. Sections were further incubated with an excess of unconjugated Fab antibody (AffiniPure Fab Fragment Goat Anti-Rabbit IgG, Jackson Immuno Research Lab, West Grove, PA) derived from the same host species as the primary antibody for 1 hour at room temperature and washed with PBS. The sections were finally incubated with the mixed second primary antibodies (anti-BrdU, anti-β-gal, anti-NKX2-1) overnight at 4°C, washed with PBS, and were incubated with the second secondary antibody (Alexa Fluor 488 goat anti-rat IgG, Dylight 650 goat anti-chicken IgY, Dylight 594 goat anti-rabbit IgG) for 1 hour at room temperature and washed with PBS. DAPI dye (Life Technology, Carlsbad, CA) was used to stain the nuclei of cells. Confocal images were obtained with a Zeiss 780 LSM (Carl Zeiss AG, Germany).

### Primary thyroid tissue slice culture

Resected thyroid was sliced in 100–150 µm thickness using McIlwain tissue chopper (Mickle Laboratory Engineering, Guildford, Surrey, United Kingdom), followed by secondary slicing at perpendicular direction in 50–100 µm thickness. Sliced small thyroid tissues were embedded in collagen gel, which was layered on the top of acellular collagen gel in a cell culture insert (BD Biosciences, Franklin Lakes, NJ). The insert with embedded thyroid slices in collagen gels was placed onto a culture dish, which was filled with DMEM/F12 containing 10% fetal bovine serum with antibiotic/antimycotic, and was placed in a tissue culture incubator under 5% CO_2_. BrdU (10 mM) was added to the medium 2 hours before harvesting whole embedded tissues on day 1, 3, and 7.

A whole collagen gel containing thyroid slices was fixed in 10% buffered formalin, dehydrated, embedded in paraffin, and sectioned at 4 µm, which was then subjected to histological and immunohistochemical analyses as described above. Thyroid sections were arbitrarily divided into central and peripheral areas, and positive cell numbers were determined by counting 10 sections prepared from a culture dish and were expressed as positive cells per 300 intrafollicular or mesenchymal cells. Statistical analysis was carried out using student *t*-test. P<0.05 was considered as statistical significance.

## Results

### R26R;TPO-Cre mice for thyroid lineage tracing

R26R;TPO-Cre mice were used to carry out thyroid lineage tracing to understand how thyroid follicular cells regenerate after thyroid damage. R26R;TPO-Cre mice produceβ-gal upon expression of the Cre recombinase, gene under control of the human thyroid peroxidase (TPO) gene promoter [26]. TPO, an enzyme required for thyroid hormone synthesis, is expressed in thyroid follicular cells [28]. The expression commences around embryonic day (E) 15 of mouse gestation at which time thyroid hormone synthesis begins [26], [29]. TPO is considered to be a thyroid follicular cell differentiation marker, and thus β-gal expression in R26R;TPO-Cre mice can be used to trace the lineage of thyroid follicular cells. In order to produce thyroid damage, the R26R;TPO-Cre mice were subjected to partial thyroidectomy (PTx) that was previously shown to serve as a model to study thyroid regeneration [30]. PTx was further combined with BrdU long label-retaining cell analysis. Long label-retaining cells serve as a surrogate marker to identify populations containing cells with stem/progenitor cell properties without the knowledge of specific markers for stem/progenitor cells present in that particular tissue [19], [21], [22], [24] [25]. The combination of these analyses allowed us to study the regeneration process of thyroid follicular cells and to obtain an insight into putative stem/progenitor cells of the thyroid.

### Appearance of Sca1 positive cells in non-follicular mesenchymal area on day 7 post-PTx

**Figure 1:**
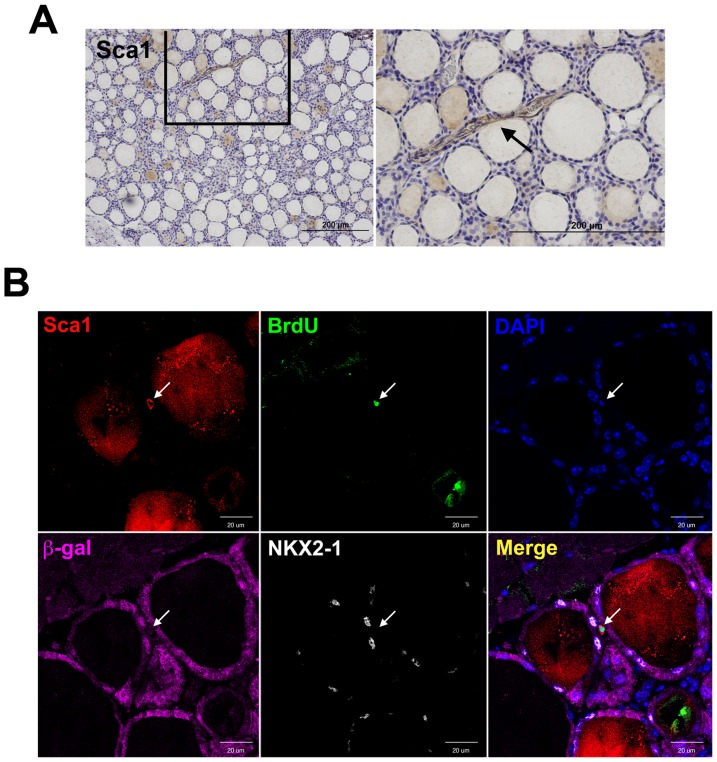
Immunohistochemistry and immunofluorescence of Sca1, BrdU, β-gal, and NKX2-1. (A) Sca1 immunohistochemistry of normal thyroid. The image on the right shows the magnified image boxed on the left. Arrow indicates positive staining (brown in color) in the vascular endothelial cells. Scale bar: 200 µm. (B) Immunofluorescence for Sca1, BrdU, DAPI, β-gal, and NKX2-1 in 7 days post-PTx thyroid. Arrow indicates a positive cell for Sca1 and BrdU, but negative for β-gal, and NKX2-1, located in the mesenchymal area. Merged image is shown on the lower right. Scale bar: 20 µm.

We previously demonstrated that thyroid SP cells exhibited stem/progenitor cells-like characteristics and approximately a half expressed Sca1 as determined by FACS analysis [1]. Thus, the Sca1(+) SP cells corresponded to ∼0.2–0.7% of total thyroid cells. Immunohistochemistry demonstrated that Sca1 immunostaining was occasionally observed in vascular endothelial cells of normal thyroid, but rarely observed in thyroid follicular cells (Figure 1A). When R26R;TPO-Cre mice were subjected to PTx in conjunction with BrdU labeling, and their thyroids were examined at 7 days post-PTx for Sca1 expression, a few Sca1(+) cells were found in non-follicular mesenchymal areas (Figure 1B). These Sca1(+) cells did not have the spindle shape typical of endothelial cells, and were also positive for BrdU, thus suggesting that Sca1(+) cells located in the non-follicular mesenchymal areas were proliferating right after PTx. We next examined whether these cells express β-gal and/or NKX2-1. NKX2-1 is a homeodomain transcription factor critical for thyroid organogenesis [31] as well as control of thyroid-specific expression of genes such as thyroglobulin [32], TPO [33], [34], and TSH receptor [35], [36]. NKX2-1 is considered as an early thyroid differentiation marker [1]. The results demonstrated that Sca1(+);BrdU(+) cells were negative for both β-gal and NKX2-1, suggesting that these cells were not of thyroid follicular cell origin.

### Co-localization of Sca1 and Krt14-expressing cells

**Figure 2:**
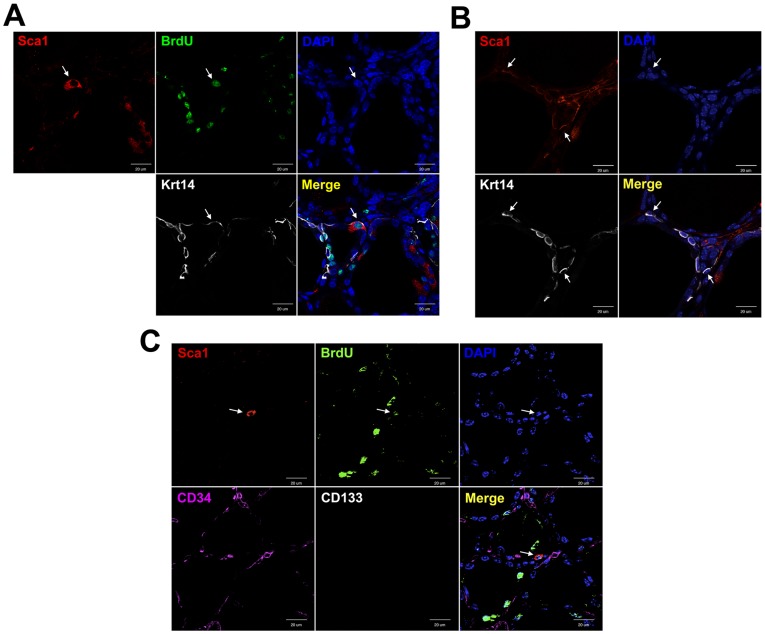
Co-expression of Sca1 and Krt14, CD34, or CD133 in the thyroid 14 days post-PTx by immunofluorescence. (A, B) Sca1(+) cells located in the follicles (A) or in the mesenchymal areas (B) co-express Krt14. Arrow indicates cells co-expressing Sca1, Krt14, and/or BrdU. (C) Sca1(+);BrdU(+) cells (shown by an arrow) do not co-express either CD34 or CD133. Merged image is shown on the lower right. Scale bar: 20 µm.

We previously demonstrated that cytokeratin 14 (Krt14) is temporarily expressed in restricted areas of thyroid after PTx [30]. Krt14 is known as a marker for liver progenitor cells [37], and is expressed in immature and/or stem/progenitor cells of taste buds [38] and prostate [39]. At 14 days post-PTx thyroid, some follicular cells co-expressed Sca1, BrdU, and Krt14 (Figure 2A), while some Sca1(+);Krt14(+) cells were found in non-follicular areas (Figure 2B). In order to determine the nature of Sca1(+) cells, immunofluorescence for CD34 and CD133 was carried out using 14 days post-PTx thyroid. Vascular endothelial cells were found as CD34 positive, while no cells were CD133 positive (Figure 2C and S1). Most importantly, Sca1(+);BrdU(+) cells were never CD34 nor CD133 positive, suggesting that Sca1(+);BrdU(+) cells are not of hematopoietic origin. When the expression of Oct3/4 as a marker for embryonic and adult stem cells [40], [41] and GATA4 as an early endoderm cell marker [2] was examined, no expression was found (Figure S2, S3). Further, when the expression of Sox 10 as a marker for neural crest derived cells was examined [42], [43], no positive signals were found anywhere, suggesting that Sca1(+);BrdU(+) cells may not be derived from neural crests (Figure S4).

### High Sca1 expression in irregular follicles with no β-gal expression at 35 days post-PTx

**Figure 3:**
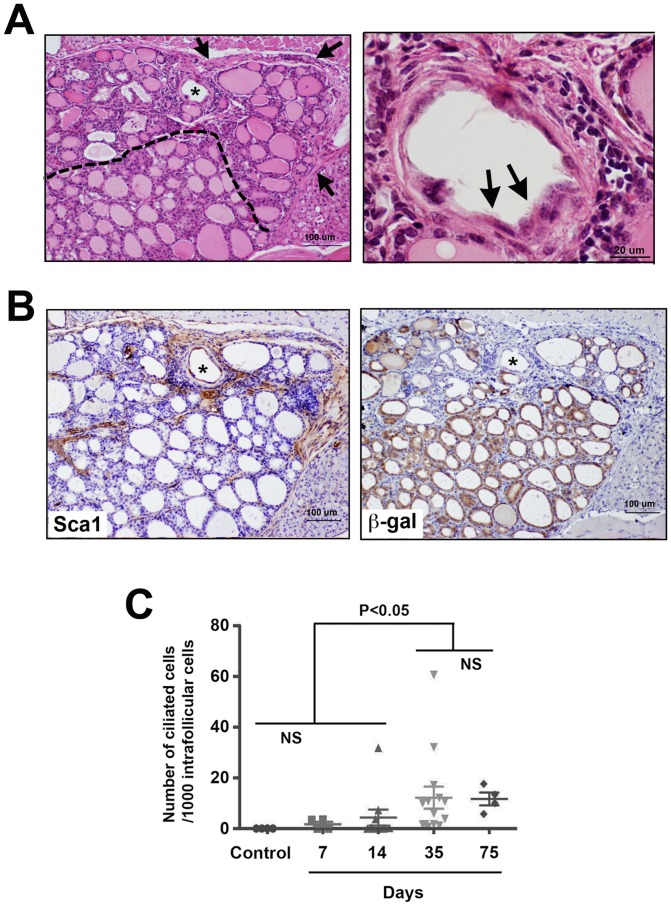
Thyroid of 35 days post-PTx. (A) H&E staining of affected areas after PTx. Left: the asterisk shows the representative follicle under repair with no colloid and in irregular shape. The dotted line demarcates the affected areas (above the line), where many irregular follicles with low or no colloid and fibrosis (arrows) are seen. Right: High magnification image of the follicle with the asterisk in A, in which many ciliated intrafollicular cells are seen (representatives shown by arrows). Scale bar: 100 µm (left), 20 µm (right). (B) Immunohistochemistry for Sca1 and β-gal shown in brown. Serial sections to that used in A were used for immunostaining. The asterisk is used to orient the sections. Scale bar: 100 µm. (C) Number of ciliated cells per 1000 intrafollicular cells in the affected area on various days post-PTx. Significant differences were found between control (no surgery), 7 days, and 14 days post-PTx thyroids vs. 35 and 75 days post-PTx thyroids. No statistically significant differences were found among control, 7 days, and 14 days post-PTx thyroids, and between 35 vs. 75 days post-PTx thyroids.

After partial thyroidectomy, some follicles in the area close to the cut edge became irregular in shape, which was most profound at day 35 post-PTx (Figure 3A). This affected area was considered under repair after damage caused by PTx. Many irregular follicles had low or no colloid inside, surrounded by fibrosis, in which ciliated follicular cells were frequently found. The number of ciliated intrafollicular cells peaked at 35 days and remained at similar numbers at day 75 post-PTx (Figure 3C). Interestingly in the affected area, high Sca1 expression was observed, particularly in non-follicular cells, whereas most of follicular cells in this area did not express β-gal (Figure 3B). Otherwise, β-gal was uniformly expressed in follicular cells lying normal follicles.

### Differentiation of Sca1-positive, β-gal negative intrafollicular cells

**Figure 4:**
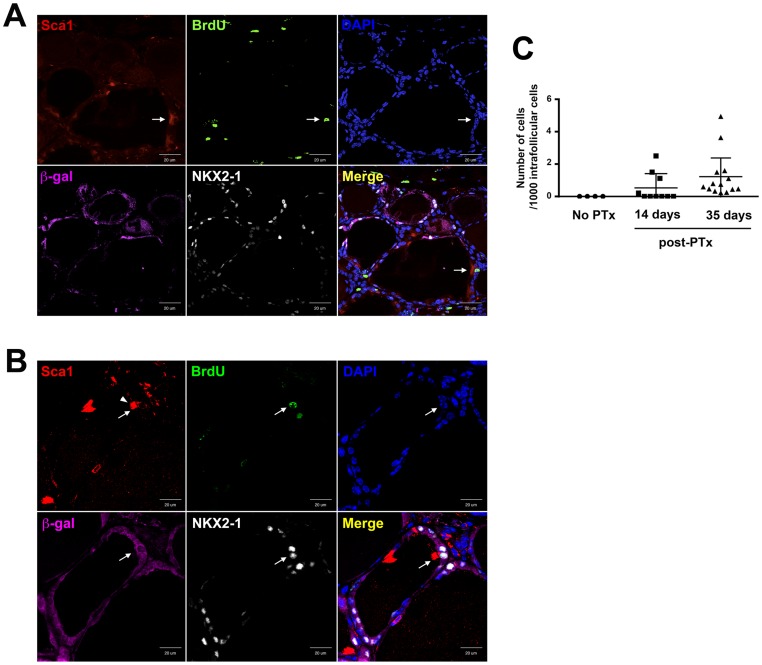
Immunofluorescence of BrdU, Sca1, NKX2-1, and β-gal in 35 and 120 days post-PTx thyroids. (A) Immunofluorescence in 35 days post-PTx thyroid. Sca1(+);BrdU(+), but β-gal(−);NKX2-1(−) cell is located in an irregular shaped follicle (shown by an arrow). (B) Immunofluorescence in 120 days post-PTx thyroid. Sca1(+);BrdU(+) cell also expresses β-gal and NKX2-1 (shown by an arrow). Arrowhead in the Sca1 panel in B indicates cilia that are strongly positive for Sca1 due to the cell having these cilia is Sca1(+). Merged image is shown on the lower right. Scale bar: 20 µm. (C) Number of Sca1(+);β-gal(−) cells in 14 and 35 days post-PTx thyroids. The number shown is based on 1000 intrafollicular cells counted. N = 7–10.

In the affected irregular follicles of 35 days post-PTx thyroids, Sca1, BrdU, NKX2-1, and β-gal expression was examined by immunofluorescence at each cell level (Figure 4A). Positive immunofluorescence for both Sca1 and BrdU was occasionally found among intrafollicular ciliated cells comprising irregular shaped follicles, which were negative for β-gal and NKX2-1. Most intrafollicular cells in the irregular shaped follicles in the affected area were NKX2-1(−);β-gal(−) at 35 days post-PTx. The presence of Sca1(+);BrdU(+);NKX2-1(−);β-gal(−) intrafollicular cells in these irregular shaped follicles suggested that the irregular shaped follicles may consist of newly formed follicular cells, which do not originate from previously differentiated follicular cells, and have not differentiated enough to express NKX2-1, resulting in no expression of β-gal. At 120 days post-PTx, some intrafollicular cells, although rarely found, were positive for all Sca1, BrdU, NKX2-1, and β-gal in mostly β-gal-expressing irregular shaped follicles (Figure 4B). These results suggested that the irregular shaped follicles may become functional follicles that express TPO, the enzyme necessary for iodination and coupling of tyrosine residues of thyroglobulin during thyroid hormone synthesis, on which β-gal expression depends. Using immunohistochemical sections of day 35 post-PTx thyroids, the number of Sca1(+);β-gal(−) cells per intrafollicular thyroid cells was counted. More cells were Sca1(+) at 35 days than 14 days post PTx. Even though, only ≤0.1% of intrafollicular thyroid cells expressed Sca1 at day 35 post-PTx that may have participated in the regeneration and/or differentiation of thyroid follicular cells after PTx (Figure 4C).

### Primary thyroid slice tissue culture as an *in vitro* model of thyroid repair

**Figure 5:**
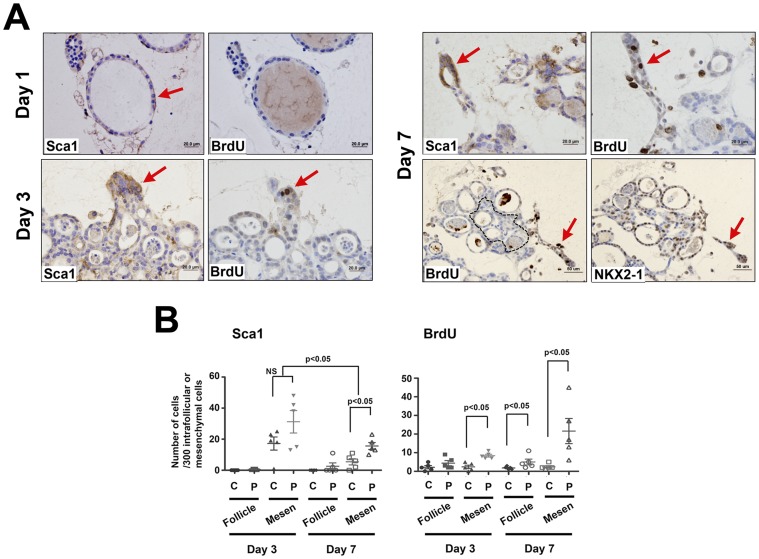
*Ex vivo* three-dimensional primary thyroid slice culture. (A) Thyroid slices were treated with BrdU two hours before harvest at 1, 3, and 7 days after the start of culture. Sections were immunostained for Sca1, BrdU, and NKX2-1 on indicated days. Serial sections were used. Red arrow shows representative Sca1, BrdU, and/or NKX2-1 positive cells. The dotted line in the panel of day 7 BrdU was arbitrarily drawn to separate the central and the peripheral area. They were used to count cell numbers shown in B. Scale bar: 20 µm (Day 1, 3, and 7 upper panel), 50 µm (Day 7 lower panel). (B) Number of Sca1(+) and BrdU(+) cells in primary thyroid slice cultures. Cell numbers were determined based on those obtained after counting 300 intrafollicular (Follicle) or mesenchymal cells (Mesen) in the central (C) or the peripheral (P) area using 10 sections prepared from a culture dish on day 3 and 7. Each dot indicates cell numbers from one culture dish.

In order to further provide evidence that Sca1(+) cells participate in thyroid follicular cell regeneration after thyroid damage, thyroids were sliced and subjected to *ex vivo* three-dimensional collagen gel tissue culture. Cultured thyroid slices were pulse-labeled with BrdU for two hours before harvest at day 1, 3, and 7 (Figure 5). Particular attention was paid to the peripheral area, assuming that cells at the peripheral area were under a similar environment to that of the area close to the cut edge of *in vivo* thyroid after PTx. On day 1, Sca1 expression was observed in the layer of cells surrounding follicular cells while no BrdU expression was found (Figure 5A). On day 3, Sca1 expression was found in non-follicular mesenchymal cells at the periphery, where some were BrdU positive. Follicles formed by day 7, in which intrafollicular cells expressed Sca1 and some were also positive for BrdU. NKX2-1 expression was found in some intrafollicular cells in newly formed follicles.

Numbers of Sca1 and/or BrdU(+) cells were separately counted for intrafollicular and mesenchymal cells in the central and peripheral areas after arbitral demarcation (Figure 5B). On both day 3 and 7, more cells in the peripheral area expressed Sca1 and BrdU as compared to the central area, supporting our hypothesis that cells in the peripheral areas of sliced thyroid resemble those found near to the cut edge of PTx thyroids. On day 7, Sca1 expression commenced in the peripheral intrafollicular cells as compared to the intrafollicular cells of the central areas although no statistical significance was found, and statistically significantly higher BrdU expression was observed. These results suggested the temporal and transitional expression pattern of Sca1, in which Sca1(+) cells appear in non-follicular mesenchymal areas after initiation of *ex vivo* culture, followed by appearance in the intrafollicular areas, which express NKX2-1. Thus, the primary thyroid slice culture appeared to recapitulate the *in vivo* process of thyroid regeneration.

## Discussion

In this study using lineage tracing and long label-retaining cell analyses, we demonstrated that Sca1(+)cells may be responsible for repair and/or regeneration of thyroid follicular cells after damage caused by PTx. While Sca1 immunopositivity was frequently found in vascular endothelial cells of normal thyroid, no Sca1 expression was found in other types of cells including those residing in mesenchymal and epithelial areas. Upon PTx, Sca1(+);BrdU(+) cells were found in the non-follicular mesenchymal areas of thyroid, which were β-gal(−);NKX2-1(−). They temporarily co-expressed Krt14; some were found in mesenchymal areas while others were found as intrafollicular cells by 14 days post-PTx. In 35 days post-PTx thyroid, Sca1(+);BrdU(+);β-gal(−);NKX2-1(−) cells were clearly found as part of follicles. At this stage, most of Sca1(+);BrdU(+) cells were β-gal(−);NKX2-1(−), while they became β-gal(+);NKX2-1(+) by 120 days post-PTx. Only a tiny fraction of ≤0.1% of total intrafollicular cells were Sca1(+) on day 35 post-PTx.

Most of newly formed Sca1(+);BrdU(+);β-gal(−) intrafollicular cells were found in the area where many irregular follicles were present after PTx, most profoundly at 35 days post-PTx. In these irregular follicles, many ciliated intrafollicular cells were observed, which also peaked at 35 days post-PTx. The irregular follicles with ciliated cells observed in the current study resemble the solid cell nests (SCN) previously described in humans [44]–[47] or a second type of follicle that was described decades ago in rodents [48], [49]. SCN is characterized as the structure having both solid cell proliferation and cyst-like structure, while the second kind of follicle is characterized by a nonhomogenous or foamy colloid, with both having ciliated cells. They are considered to be the embryonic remnants of the ultimobranchial body (UBB) that migrates from the fourth pharyngeal pouch, fuses with the thyroid primordium around E 14.5 of mouse gestation, and ultimately gives rise to the calcitonin-producing C cells [50]–[52]. The SCN, the cystic vesicular structure of UBB, has centrally located p63-negative undifferentiated cells, surrounded by a cluster and/or single layer of p63(+) cells, displaying a basal/stem cell phenotype [45], [52], [53]. The nature of undifferentiated cells in the SCN and the significance of p63 expression in this structure are not known. Previously, the involvement of SCN as the source for follicular cells and C cells was suggested [52], [54]–[56]. In the current study, we observed; 1) after PTx, intrafollicular cells in irregular follicles with ciliated cells are Sca1(+);β-gal(−), 2) the number of Sca1(+);β-gal(−) cells increases at 35 days post-PTx, 3) Sca1(+);β-gal(−) cells undergo differentiation to become differentiated follicular cells, and 4) ciliated cells in the irregular follicles increase in number, and reach maximal numbers at 35 days post-PTx. Based on these facts, it is tempting to consider the possible involvement of SCN in thyroid regeneration. Further experiments are required to address these questions.

In the *ex vivo* study using primary thyroid slice culture in collagen gel, the higher proliferation was found in the peripheral area as compared to the central area, which we hypothesized was due to sudden loss of tissues, a condition similar to that found near to the cut edge of PTx. However, the possibility cannot be excluded that the higher proliferation at the peripheral area was simply due to more access to oxygen at the periphery than the central area. Toda et al also reported that the growth and folliculogenesis occurred at five fold higher levels at the periphery in collagen gel thyroid cell primary cultures [57]. In any event, the apparent transitional expression of Sca1 from mesenchymal to intrafollicular cells was recapitulated in *ex vivo* primary thyroid slice collagen gel culture studies. With *in vivo* and *ex vivo* results together and the fact that Sca1(+) cells temporarily co-express Krt14, it is tempting to speculate that Sca1(+) cells while residing in mesenchymal areas may start acquiring epithelial features, while they retain the capacity to migrate into follicles [58]. Sca1(+) cells found in non-follicular mesenchymal area at 7–14 days after PTx did not express Oct3/4, a classical marker for stem cells, GATA4, an early endoderm cell marker, nor Sox10, a marker for neural crest-derived cells. The latter suggests that Sca1(+) cells appeared after PTx may not be derived from neural crest. However, the possibility remains that expression of all these markers could be very temporal and we just missed detecting them. Where Sca1(+) mesenchymal cells come from, whether Sca1(+) mesenchymal cells actually migrate into follicles, and whether Sca1 can serve as a marker for thyroid stem/progenitor cells require further experiments.

**Figure 6:**
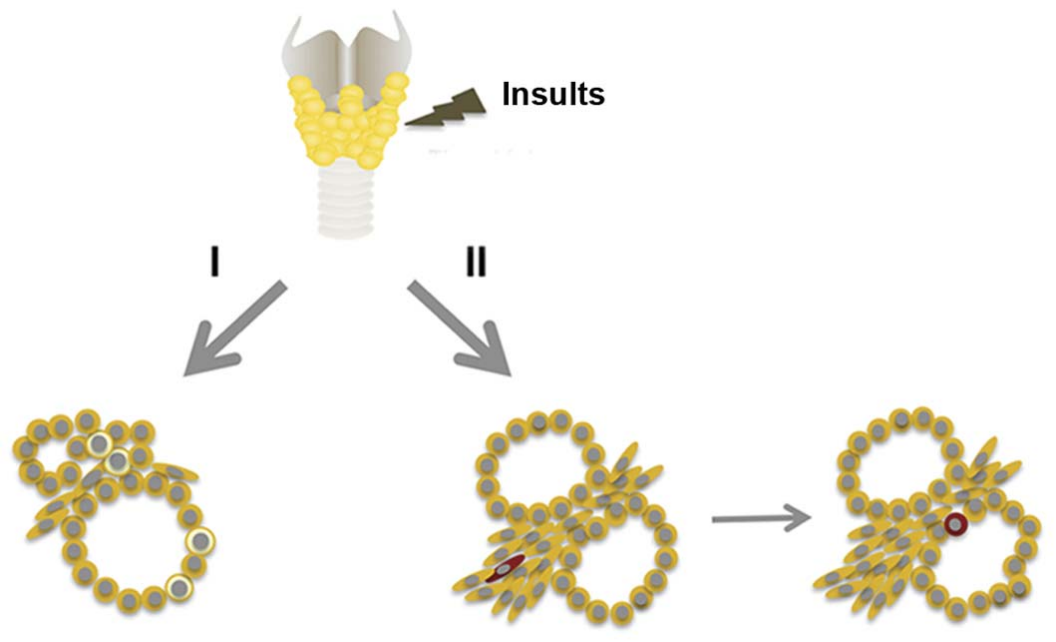
Model depicting two independent regenerative pathways that may operate in thyroid after damage caused by various insults such as chemicals, radiation, and surgery. Pathway I: clear immature cells with characteristics of C cells or follicular cells appear at ∼7–14 days post PTx in mice [30]. Clear cells are shown by pale yellow color. Pathway II: Sca1(+) cells appear in mesenchymal areas at ∼7–14 days post PTx, followed by the appearance of Sca1(+) cells in follicles at ∼14–35 days post PTx. Sca1(+) cell is shown by dark-red color.

We recently described PTx altered cells and a pattern of gene expression in the thyroid that resembled those found during thyroid development and cancer when examined at 2 weeks post-PTx [30]. These alternations further involved the appearance of many clear immature cells. Electron microscopic analysis revealed that the clear cells may have been previously C cells or follicular cells that were altered after partial thyroidectomy to become immature cells. Alternatively, they were immature cells that might have been derived from stem/progenitor cells of thyroid. These alterations were observed 1–2 weeks after PTx. In the current study, Sca1(+) non-vascular mesenchymal cells were found ∼1 week after PTx, while Sca1(+) intrafollicular cells were found at 14–35 days post PTx and thereafter. The most profound changes were observed 35 days post-PTx. We currently do not know the relationship of Sca1(+) cells identified in the current study to clear immature cells identified in the previous study. Perhaps multiple layers of events may occur after PTx to assure successful regeneration as diagramed in Figure 6.

In conclusion, Sca1(+) mesenchymal cells were identified that may be involved in the process of thyroid differentiation into mature follicular cells after PTx. The *ex vivo* study using primary thyroid slice culture in collagen gel recapitulated the *in vivo* process. These studies will facilitate research on thyroid stem/progenitor cells and their roles in thyroid diseases, particularly thyroid carcinomas, and thyroid regenerative medicine.

## Supporting Information

Figure S1 **Immunohistochemistry (A) and immunofluorescence (B, C) for CD34 and CD133 using normal adult mouse kidney as positive control (A, left panel, B, C) and without primary antibody as negative control (A, right panel).**
(TIF)

Figure S2 **Immunofluorescence for Oct3/4 using E12.5 mouse embryo gonad as positive control (A) and day 14 post-PTx thyroid (B).** Thyroid did not have any positive signal for Oct3/4 expression.
(TIF)

Figure S3 **Immunofluorescence for GATA4 using E12.5 mouse embryo developing heart as positive control (A), that without antibody as negative control (B), and day 14 post-PTx thyroid (C).**
(TIF)

Figure S4 **Immunohistochemistry (A) and immunofluorescence (B, C) for Sox10.** (A, B) Adult mouse brain was used as a positive control. Sox10 negative control was without primary antibody (A, lower right panel). (C) Post-PTx thyroid did not show any Sox10 positive fluorescence (cell indicated by an arrow).
(TIF)
