# Supplementary figures and images for: Stem Cell Antigen 1-Positive Mesenchymal Cells Are the Origin of Follicular Cells during Thyroid Regeneration
Source: PLoS One. 2013 Nov 21;8(11):e80801. doi: 10.1371/journal.pone.0080801 (PMC3836768; doi:10.1371/journal.pone.0080801)

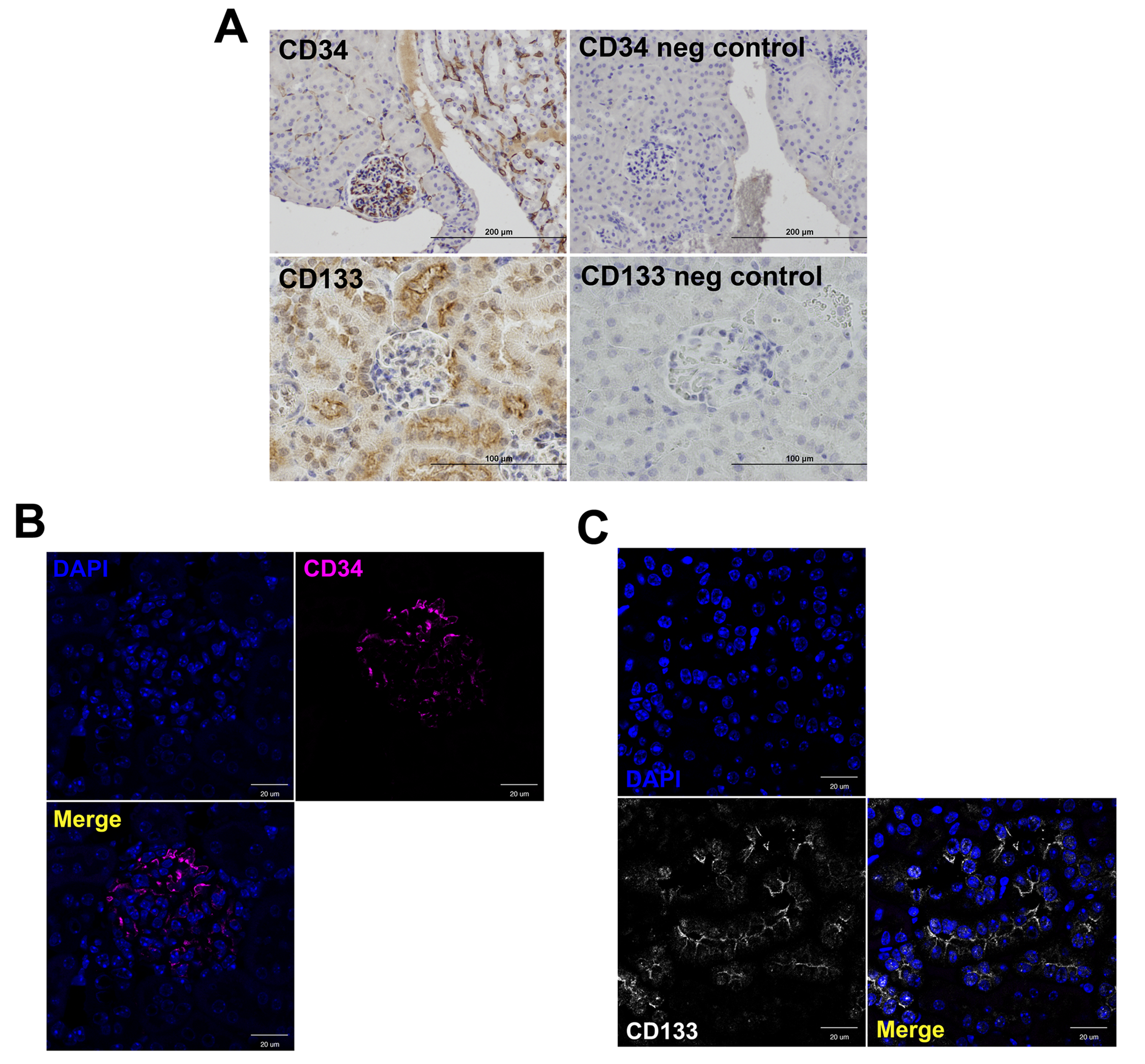

Supplement: Figure S1 — Immunohistochemistry (A) and immunofluorescence (B, C) for CD34 and CD133 using normal adult mouse kidney as positive control (A, left panel, B, C) and without primary antibody as negative control (A, right panel). (TIF) [file pone.0080801.s001.tif]

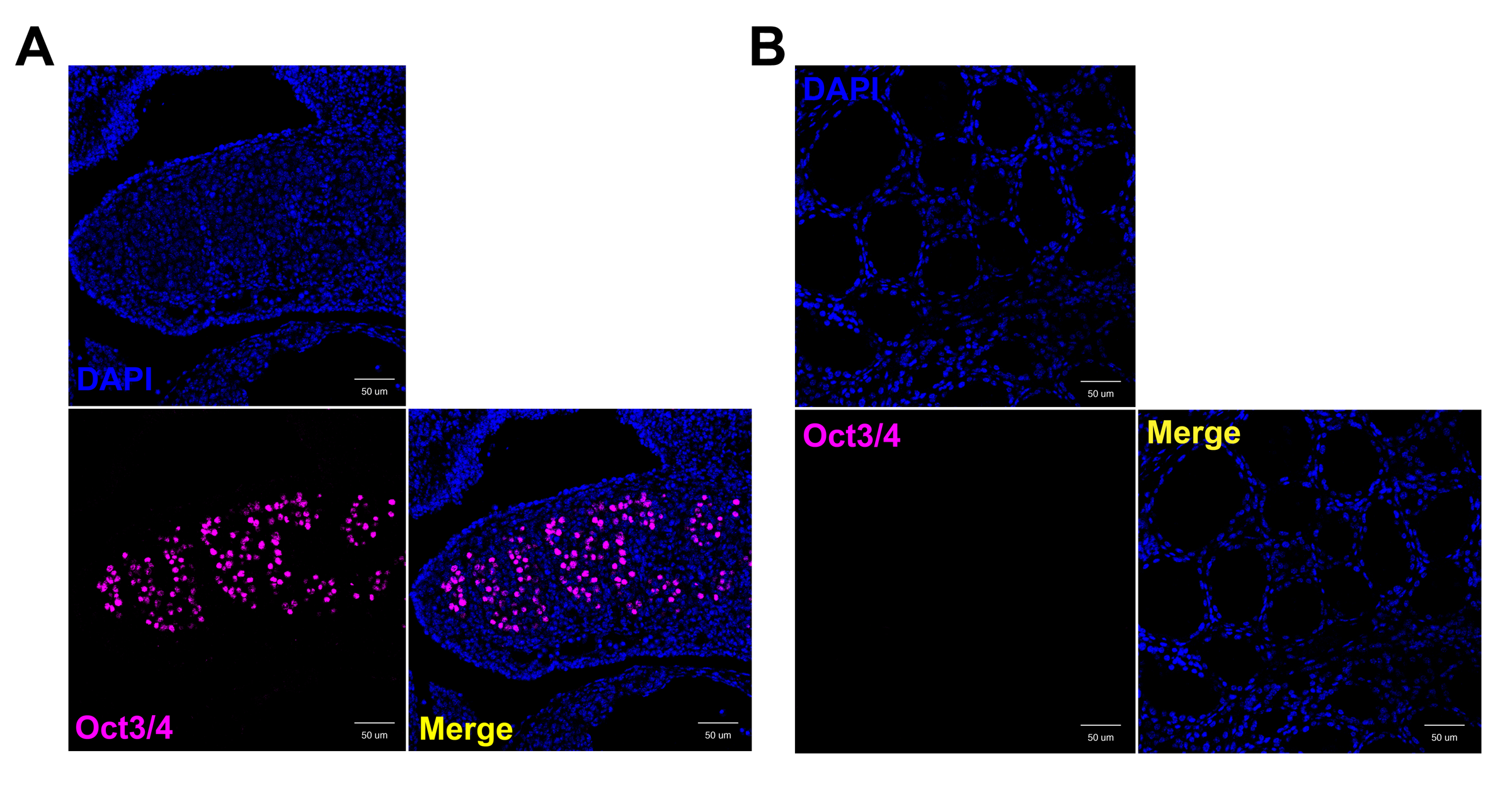

Supplement: Figure S2 — Immunofluorescence for Oct3/4 using E12.5 mouse embryo gonad as positive control (A) and day 14 post-PTx thyroid (B). Thyroid did not have any positive signal for Oct3/4 expression. (TIF) [file pone.0080801.s002.tif]

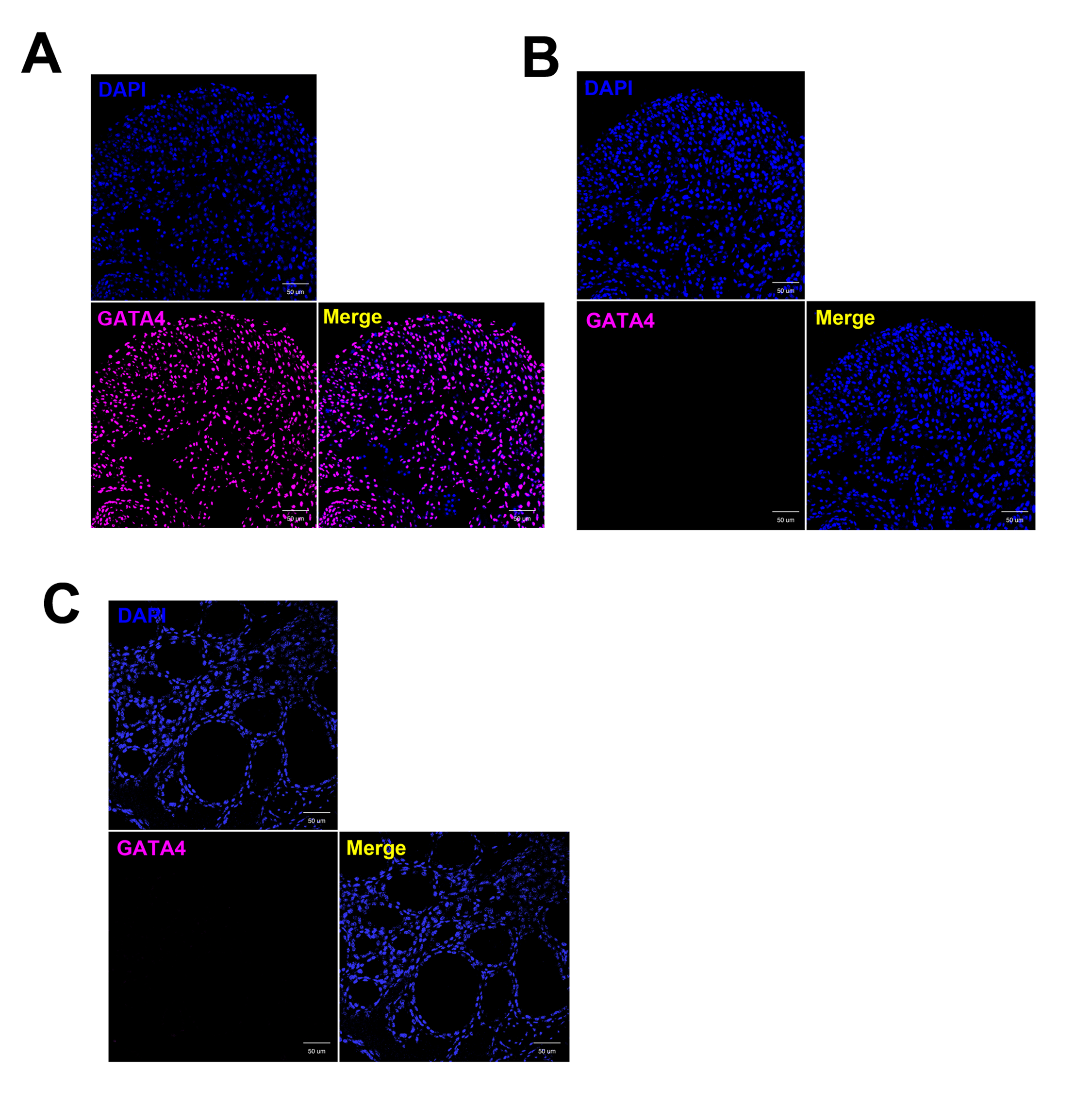

Supplement: Figure S3 — Immunofluorescence for GATA4 using E12.5 mouse embryo developing heart as positive control (A), that without antibody as negative control (B), and day 14 post-PTx thyroid (C). (TIF) [file pone.0080801.s003.tif]

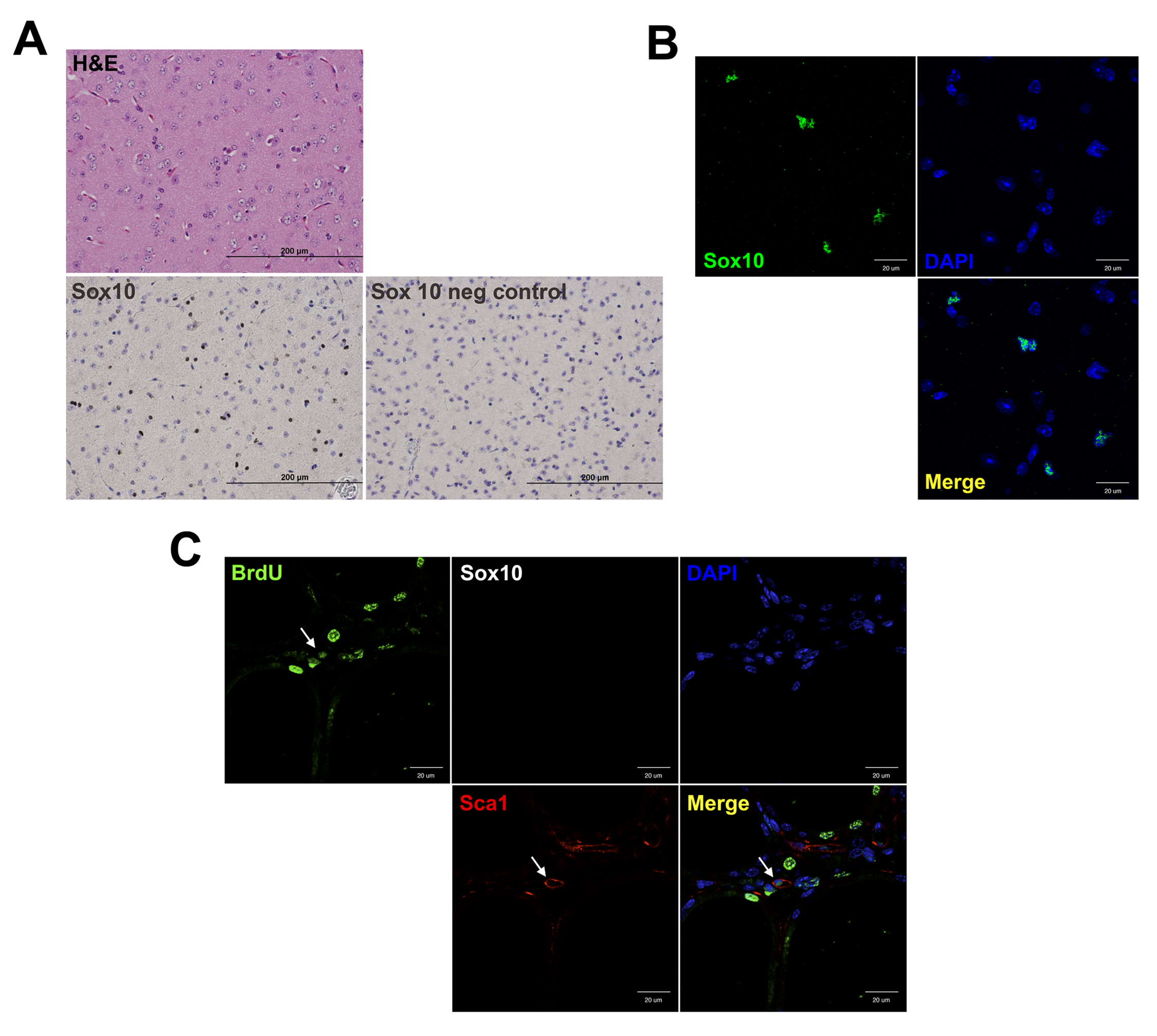

Supplement: Figure S4 — Immunohistochemistry (A) and immunofluorescence (B, C) for Sox10. (A, B) Adult mouse brain was used as a positive control. Sox10 negative control was without primary antibody (A, lower right panel). (C) Post-PTx thyroid did not show any Sox10 positive fluorescence (cell indicated by an arrow). (TIF) [file pone.0080801.s004.tif]
